# Genomic Characterization of H14 Subtype Influenza A Viruses in New World Waterfowl and Experimental Infectivity in Mallards (*Anas platyrhynchos*)

**DOI:** 10.1371/journal.pone.0095620

**Published:** 2014-05-01

**Authors:** Andrew M. Ramey, Rebecca L. Poulson, Ana S. González-Reiche, Daniel R. Perez, David E. Stallknecht, Justin D. Brown

**Affiliations:** 1 US Geological Survey, Alaska Science Center, Anchorage, Alaska, United States of America; 2 Southeastern Cooperative Wildlife Disease Study, College of Veterinary Medicine, Department of Population Health, The University of Georgia, Athens, Georgia, United States of America; 3 Department of Veterinary Medicine, University of Maryland College Park, Virginia-Maryland Regional College of Veterinary Medicine, College Park, Maryland, United States of America; 4 Centro de Estudios en Salud, Universidad del Valle de Guatemala, Guatemala City, Guatemala; University of Minnesota, United States of America

## Abstract

Recent repeated isolation of H14 hemagglutinin subtype influenza A viruses (IAVs) in the New World waterfowl provides evidence to suggest that host and/or geographic ranges for viruses of this subtype may be expanding. In this study, we used genomic analyses to gain inference on the origin and evolution of H14 viruses in New World waterfowl and conducted an experimental challenge study in mallards (*Anas platyrhynchos*) to evaluate pathogenicity, viral replication, and transmissibility of a representative viral strain in a natural host species. Genomic characterization of H14 subtype IAVs isolated from New World waterfowl, including three isolates sequenced specifically for this study, revealed high nucleotide identity among individual gene segments (e.g. ≥95% shared identity among H14 HA gene segments). In contrast, lower shared identity was observed among internal gene segments. Furthermore, multiple neuraminidase subtypes were observed for H14 IAVs isolated in the New World. Gene segments of H14 viruses isolated after 2010 shared ancestral genetic lineages with IAVs isolated from wild birds throughout North America. Thus, genomic characterization provided evidence for viral evolution in New World waterfowl through genetic drift and genetic shift since purported introduction from Eurasia. In the challenge study, no clinical disease or lesions were observed among mallards experimentally inoculated with A/blue-winged teal/Texas/AI13-1028/2013(H14N5) or exposed via contact with infected birds. Titers of viral shedding for mallards challenged with the H14N5 IAV were highest at two days post-inoculation (DPI); however shedding was detected up to nine DPI using cloacal swabs. The distribution of viral antigen among mallards infected with H14N5 IAV was largely restricted to enterocytes lining the villi in the lower intestinal tract and in the epithelium of the bursa of Fabricius. Characterization of the infectivity of A/blue-winged teal/Texas/AI13-1028/2013(H14N5) in mallards provides support for similarities in viral replication and shedding as compared to previously described waterfowl-adapted, low pathogenic IAV strains in ducks.

## Introduction

Influenza A viruses (IAVs) infect humans and numerous domestic and wild mammals and birds; however, most of the antigenic diversity is maintained in wild waterbirds of the orders *Charadriiformes* and *Anseriformes* [1]. This antigenic diversity includes 16 hemagglutinin (HA) and nine neuraminidase (NA) subtypes in avian hosts [1]–[3]. The surface glycoproteins (HA and NA) of IAVs, which are used to designate viral subtypes, enable binding of virions to host cells and facilitate release of progeny viruses after replication [1]. Results from extensive sampling of wild waterbirds suggest that some subtypes may have restricted geographic [2] or host ranges [3]–[5] while others have more global distribution and have been detected in a diversity of taxa [1], [6]–[7]. Factors affecting the current geographic and host ranges of specific subtypes remain unclear.

The H14 HA subtype was first identified in IAVs isolated from samples collected from mallards (*Anas platyrhynchos*) in the former Soviet Union in 1982 [8]. Detections of this viral subtype had not been reported again for nearly 30 years when, in 2010, H14 HA viruses were isolated from samples collected from long-tailed ducks (*Clangula hyemalis*) and a white-winged scoter (*Melanitta fusca*) harvested in Wisconsin [9]. Subsequently, H14 viruses were identified from samples collected from a northern shoveler (*Anas clypeata*) in California in 2011 [10] and several blue-winged teal (*Anas discors*) sampled in Guatemala in 2011 and in Texas in 2013 [11]. It is unclear if IAVs of the H14 HA subtype have been recently introduced into North America or, alternatively, that this subtype has been present but undetected in the New World for an extended period of time [12]. Recent repeated isolation of H14 IAVs in the New World provides evidence that viruses of this HA subtype may now be maintained in North American waterfowl and that host and/or geographic range may be expanding.

The goal of this study was to compare genomic characteristics and infectivity of H14 subtype IAVs to viruses of other HA subtypes commonly detected in New World waterfowl (e.g. H3). Specific objectives were: (1) to gain inference on the origin and evolution of H14 viruses in the New World through genomic analyses and (2) to evaluate pathogenicity, viral replication, and transmissibility of a representative viral strain in natural host species through an experimental challenge study of mallards (*Anas platyrhynchos*). Mallards were selected because they represent the most abundant waterfowl species in North America [13] and are frequently infected with IAVs [14]–[16]. Results from this investigation will be useful for understanding the natural history and evolution of H14 viruses in the New World, as well as gaining inference on how viruses of this subtype may be maintained and spread.

## Materials and Methods

### Ethics Statement

Live capture and sampling of blue-winged teal were approved by the U. S. Geological Survey Alaska Science Center Institutional Animal Care and Use Committee (IACUC; AUP#: 2012-2) and conducted under the authority of United States Department of the Interior Federal Bird Banding Permit #09072. All procedures used in the raising of the mallards and during the inoculation study were approved by the IACUC at the University of Georgia (UGA; AUP#: A2013 05-021-Y1-A0).

### Genomic Characterization

Three H14 subtype IAVs were selected for genomic characterization as part of the current study. Viruses were isolated from paired swab samples collected from blue-winged that were either hunter-harvested in Guatemala in 2011 (A/blue-winged teal/Guatemala/CIP049H105-15/2011(H14N3) and A/blue-winged teal/Guatemala/CIP049H106-62/2011(H14N6)) or live-captured in Texas in 2013 (A/blue-winged teal/Texas/AI13-1028/2013(H14N5)). Viruses were isolated in embryonated eggs and RNA extracted from allantoic fluid as previously reported [11].

For isolates from Guatemala, complete genome sequences were obtained by pyrosequencing in a 454 GS Junior system using previously described protocols for viral RNA extraction [17] and preparation of barcoded SISPA libraries [18]–[19]. Briefly, non-viral nucleic acids were removed through nuclease treatment (Benzonase (125U), DNase I (10U), RNase T1 (200U) and RNase A (50U)) for 30 min at 37°C. Viral RNA was then extracted using Trizol-LS (Invitrogen, Grand Island, New York, USA) in combination with RNeasy Mini spin columns (Qiagen, Gaithersburg, Maryland, USA). For the SISPA reaction, first and second strands were transcribed and amplified with barcoded random hexamers and influenza A specific primers. Library fragment end repair, quantification, emulsion PCR and sequencing were performed according to 454 GS Junior system method manuals omitting the nebulization step. The libraries were generated from 125 ng of DNA and size selected with a 0.7× SPRI. Libraries were pooled in equimolar amounts prior emulsion PCR and sequencing. Sequence assembly was performed with the 454 GS Junior Newbler software (version 2.7; Roche). GS De Novo Assembler was used to assemble initial contigs. Contigs were used to perform BLAST searches, and reference genomes were downloaded from NCBI Genbank database. Sequence reads were re-assembled against reference genomes using the GS Reference Mapper software. Contig assembly was refined with multiple rounds of reference mapping with default parameters to obtain final consensus sequences for each virus genome. Over 30,000 reads were generated for each virus with an average depth >800 in final alignments for both viruses.

IAV isolate A/blue-winged teal/Texas/AI13-1028/H14N5 was sequenced using Sanger sequencing methodology as reported by Ramey et al. [20]. Briefly, all eight RNA segments were amplified with the one-step RT PCR kit (Qiagen, Inc., Valencia, California, USA) using a combination of previously published primers [15], [21]–[26] and four primers for the H14 HA gene designed specifically for this study (H14 3F: CAAAAGCAGGGGAAAATGAT, H14 789R: CCCCTGGGTTTACTAGRGTCC, H14 739F: GGAATCAGAGCGGCAGARTA, and H14 1640R: TGACATGGAGAAAGAAATCCA). PCR products were gel purified and extracted using the QIAquick gel extraction kit (Qiagen, Inc., Valencia, California, USA) or treated with ExoSap-IT (USB Inc., Cleveland, Ohio, USA) without additional purification before sequencing. Cycle sequencing was performed with identical primers used for PCR along with BigDye Terminator version 3.1 mix (Applied Biosystems, Foster City, California, USA). Samples were analyzed on an Applied Biosystems 3730×l automated DNA sequencer (Applied Biosystems, Foster City, California, USA). Sequences were assembled and edited with Sequencher version 5.1 (Gene Codes Corp., Ann Arbor, Michigan, USA).

Partial or complete open reading frames were sequenced for all 24 individual gene segments from the three H14 IAV isolates from Guatemala and Texas. For isolate A/blue-winged teal/Guatemala/CIP049H105-15/2011(H14N3), two variants of the PA gene were identified and both sequences resolved. Evidence of co-infection was not detected at the other seven gene segments for this isolate. NCBI GenBank accession numbers for H14 IAV isolates sequenced as part of this study are: KJ195665–KJ195681 (Guatemala isolates) and KF986851–KF986858 (Texas isolate).

Genetic comparisons were made among homologous gene segments for isolates sequenced as part of this study and H14 HA subtype IAVs for which genomic information was available on the NCBI GenBank database (accessed 13 March 2014). Similarity among IAVs was assessed by first aligning consensus sequences using Sequencher version 5.1 and cropping consensus sequences for each viral strain to a common length (reported in nucleotide positions) per gene segment: PB2 (2,254), PB1 (2,261), PA (2,186), HA (1,650), NP (1,493), NA (1,410–1,415), M (942), and NS (855). Identity values were then calculated among homologous gene segments for all H14 HA IAVs using MEGA version 5.1 [27]. Similarity of the NA gene was only assessed among isolates of the same NA subtype.

The origin and evolution of the H14 HA gene of IAVs was assessed through phylogenetic analysis by comparing nucleotide sequences for HA genes sequenced as part of this study with H14 HA genes available on the NCBI GenBank database (accessed 13 March 2014) and representative H4 HA subtype lineages (Table S1). H4 HA subtype lineages were included in this analysis as this subtype has been previously shown to be the most closely related to H14 [7]–[8], [12]. Sequences were aligned using Sequencher version 5.1 and cropped to a common alignment length of 1,690 nucleotide positions. A maximum-likelihood tree was constructed with MEGA version 5.1 using the Nucleotide: nearest-neighbor-interchange methodwith 10,000 bootstrap replicates. Ancestry of H14 HA genes from Guatemala and Texas isolates was inferred based on position of nucleotide sequences relative to previously characterized sequences obtained from NCBI GenBank.

Phylogenetic analysis was also conducted for each of the six internal gene segments (PB2, PB1, PA, NP, M, and NS) of H14 HA subtype isolates to assess whether gene segments were of Eurasian, North American, or South American ancestry. This analysis was not conducted for surface glycoproteins (HA and NA) as the H14 HA gene has not yet been detected in the New World south of Guatemala and few reference sequences exist for South American IAV lineages for most NA subtypes. Phylogenies were constructed for each internal gene segment by comparing nucleotide sequences for H14 HA subtype IAVs to representative lineages of Eurasian, North American, and South American ancestry (Table S1) using MEGA version 5.1 as previously reported. Reference sequences were selected to cover a broad geographic distribution of collection locations from each continent and restricted to samples collected from 2000–2012. The continental clade in which nucleotide sequences for H14 HA subtype IAV gene segments were nested was inferred as the source of ancestral origin. Alignment lengths for internal gene segments used to create phylogenies were as follows (reported in nucleotide positions): PB2 (2,231), PB1 (2,254), PA (2,153), NP (1,435), M (943), and NS (854).

To gain inference on possible ancestral origins of the NA gene from blue-winged teal isolates from Guatemala and Texas, nucleotide sequences (1405–1464 bp) for this gene segment were compared to sequences on NCBI GenBank using the nucleotide BLAST function (accessed 6 January 2014). High nucleotide identity (i.e. >95%) to IAV strains previously isolated from samples collected in Eurasian, North American, or South American was inferred as weak evidence for possible ancestral origin of NA gene segments.

### Experimental Challenge

Eleven mallards were obtained from a commercial waterfowl breeder (McMurray Hatchery, Webster City, Iowa, USA) when they were approximately 2-days-old and cared for under indoor confinement at UGA (Athens, Georgia, USA). At 4-weeks, the birds were transferred to a biosafety level (BSL) –2 enhanced facility at the UGA. During the challenge study, ducks were group-housed by exposure category (H14 IAV-challenged or sham-inoculated) in biocontainment isolation units ventilated under negative pressure with high efficiency particulate air filters on intake and exhaust.

A first passage of A/blue-winged teal/Texas/AI13-1028/2012 (H14N5) was used as the challenge virus. Viral stock was propagated and titrated in 9- to 11-day-old specific pathogen free (SPF) embryonated chicken eggs [28]. The viral inoculum was prepared by diluting the infective amnioallantoic fluid in sterile brain-heart-infusion (BHI) media to yield a final titer of 10^6^ EID_50_/0.1 ml (single-bird inoculum; back titer: 10^5.6^ EID_50_/0.1 ml). The sham-inoculum consisted of sterile BHI media.

Immediately prior to inoculation (0 day post-inoculation (DPI)), blood samples and oropharyngeal and cloacal swabs were collected from all 11 mallards to confirm they were serologically and virologically negative for IAV, respectively. Six of the mallards were inoculated with 0.1 mL of the H14N5 virus inoculum by the intranasal route (IN; into the choanal cleft) and three were IN inoculated with 0.1 mL of the sham-solution. At 2 DPI, two of the H14N5-inoculated and one of the sham-inoculated mallards were humanely euthanized by CO_2_ inhalation and full necropsies were performed. At necropsy, tissue samples were collected from trachea, lung, air sacs, esophagus, proventriculus, duodenum, jejunum, ileum, colon, ceca, bursa of Fabricius, liver, spleen, heart, pectoral muscle, brain, adrenal gland, pancreas, and kidneys, and placed in 10% neutral buffered formalin for histopathologic examination. Also at 2 DPI, two naïve mallards were placed into the same isolator unit as the remaining four H14N5-inoculated mallards to evaluate intraspecies contact transmission. The two contact-exposed mallards were humanely euthanized at 2 day post-contact (DPC) and necropsies were performed and tissues were collected for histopathologic examination as described above. The trial was terminated at 14 DPI at which time all remaining mallards were humanely euthanized as previously described.

Tissues collected during necropsy from H14N5-inoculated, contact-exposed, and sham-inoculated mallards were fixed in 10% neutral buffered formalin for approximately 72 hours, routinely processed, and embedded in paraffin. Sections were cut at 5 µm and stained with hematoxylin and eosin. Duplicate sections were cut and also immunohistochemically-stained using a commercial mouse monoclonal antibody to the NP of influenza A virus at a 1∶1000 dilution (Biodesign International, Sako, Maine, USA) using a published procedure [29].

During the trial all mallards were monitored twice daily for behavioral changes or overt signs of disease. Oropharyngeal and cloacal swabs were collected from H14N5- and sham-inoculated mallards on 0 to 7 and 9 DPI, and from contact-exposed mallards on 0 to 2 DPC. Swab samples were immediately placed in individual cryogenic vials (Corning Inc., Corning, New York, USA) containing 2.0 mL of viral transport media (BHI media supplemented with 250 µg/ml gentamicin, 500 µg/ml kanamycin, 1,000 µg/ml, streptomycin, 1,000 units/ml penicillin G, and 25 µg/ml amphotericin B). Blood samples were collected from all mallards on 0 DPI/DPC and immediately after the ducks were euthanized. Blood samples were placed into serum separator tubes (Becton, Dickinson and Company, Franklin Lakes, New Jersey, USA) and centrifuged at 1500×g for 15 minutes to harvest serum.

Oropharyngeal and cloacal swabs collected during the trials were stored at −70°C until testing was performed. Virus isolation in 9- to 11-day-old, SPF embryonated chicken eggs was performed on all oropharyngeal and cloacal swabs collected during the trial [28]. Oropharyngeal and cloacal swabs collected from H14N5-inoculated mallards on 2, 4, and 6 DPI and from contact-exposed mallards on 2 DPC were also titrated in 9- to 11-day-old, SPF embryonated chicken eggs [28]. Viral titers were calculated using the methodology described by Reed and Muench [30] and reported in log_10_ EID_50_/mL.

Serum samples were stored at −20°C until testing was performed. All samples collected during the trials were tested for antibodies directed against the influenza nucleoprotein (NP) using a commercial blocking enzyme-linked immunosorbent assay (bELISA; FlockChek AI MultiS-Screen antibody test kit; IDEXX Laboratories, Westbrook, Maine, USA) according to the manufacturer’s instructions. Additionally, all serum samples were tested for antibodies directed against the H14 HA surface protein using a virus microneutralization assay (VN).

Antigen (A/blue-winged teal/Texas/AI13-1028/2013(H14N5)) for VN was prepared in Maden Darby Canine Kidney cells (MDCK; ATCC, Manassas, Virginia, USA). During virus propagation, and in all VN test procedures, cells were maintained in minimal essential media (MEM; Sigma-Aldrich, St. Louis, Missouri, USA) containing TPCK-trypsin (final concentration of 1 µg/ml; Worthington Biochemical Corporation, Lakewood, New Jersey, USA) and antibiotics (final concentration 100 units penicillin, 0.1 mg streptomycin, and 0.25 ug amphotericin B/ml; Sigma-Aldrich, St. Louis, Missouri, USA). Antigen was stored at −80°C until used. For antibody testing, sera were diluted 1∶10 in MEM and heat inactivated at 57°C for 30 minutes. Each serum sample was further diluted two-fold in MEM on a 96 well v-bottom plate (final volume or 25 µl at dilutions 1∶20 to 1∶640). An additional well for each serum sample served as a serum control to determine potential toxicity. A positive control well using chicken antisera to H14N5 (A/mallard/Gurjev/263/1982 (H14N5)) provided by the National Veterinary Services Laboratory, Animal and Plant Health Inspection Agency, U. S. Department of Agriculture, and a negative control well using MEM were also included. The H14 antigen (25 µl containing 100 median tissue culture infective doses (10^2.0^ TCID_50_)) was added to each well, not including the serum control wells which received 25 µl MEM. Plates were incubated for 2 hr at room temperature after which 25 µl from each well was transferred to a second 96-well tissue culture plate with a confluent monolayer of MDCK cells. Prior to transfer, the tissue culture plate containing the MDCK cells was washed two times with DPBS (Sigma-Aldridge, St. Louis, Missouri, USA) and 150 µl of trypsin supplemented MEM was added to each well. The Inoculated tissue culture plate was incubated at 5% CO_2_ at 37 C and was visually read at 48–72hr. A positive well contained no evidence of cytopathic effect. For the test result to be considered valid, all controls (serum, positive, and negative) had to meet their expected negative or positive status. In addition, based on back titration in MDCK cells, the viral titer of the antigen had to fall within 10^1.66^ and 10^2.33^ TCID_50_/25 µl. Sera were considered positive, if complete neutralization was observed at the 1∶20 serum dilution.

## Results

### Genomic Characterization

IAVs isolated from blue-winged teal sampled in Guatemala and Texas shared identity of up to 99.5% for individual gene segments (Table S2); however, no two isolates shared more than 89.7% identity across seven gene segments compared. Similarity was not calculated among the NA genes for isolates from Guatemala and Texas as isolates were different NA subtypes. When isolates from Guatemala and Texas were compared to other H14 HA subtype IAV isolates, none of the strains from Guatemala or Texas shared more than 92.0% identity across all gene segments with previously reported H14 HA subtype IAVs (Table S2). Isolate A/blue-winged teal/Guatemala/CIP049H105-15/2011(H14N3) was ≥92.0% similar to A/long-tailed duck/Wisconsin/10OS4225/2010(H14N6) and A/mallard/Wisconsin/10OS3941/2010(H14N6) at seven gene segments with regard to nucleotide sequence; however, the isolate from Guatemala had a different NA subtype than those from Wisconsin (Table S2). Among all H14 HA subtype strains compared, the two isolates with the highest shared nucleotide identity (≥99.8% at all gene segments) were A/long-tailed duck/Wisconsin/10OS3918/2010(H14N8) and A/white-winged scoter/Wisconsin/10OS3922/2010(H14N8) (Table S2).

**Figure 1:**
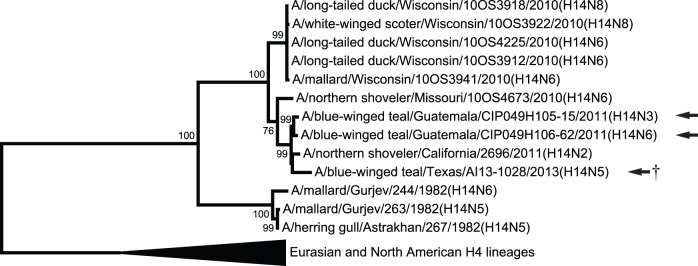
Maximum likelihood phylogenetic tree showing inferred relationship among nucleotide sequences for the hemagglutinin gene of influenza A viruses of the H14 subtype. Bootstrap support values ≥70 are shown. Isolates from Guatemala and Texas genetically characterized as part of the current study are indicated with an arrow. The isolate used to experimentally inoculate mallards is identified with a dagger (†).

**Figure 2:**
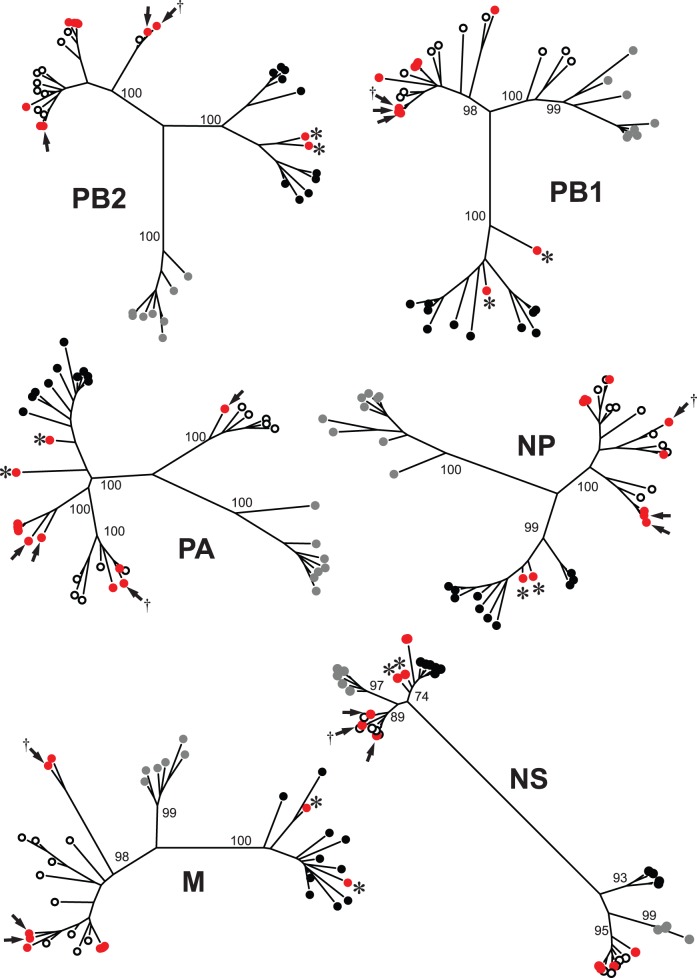
Maximum likelihood phylogenetic trees showing inferred relationship among nucleotide sequences for the internal gene segments for influenza A viruses of the H14 subtype (red circles) and reference sequences from viruses isolated from wild birds in Eurasia (black circles), North America (white circles), and South America (gray circles). Bootstrap support values for continentally affiliated clades are shown. Isolates from Guatemala and Texas genetically characterized as part of the current study are indicated with an arrow. The isolate used to experimentally inoculate mallards is identified with a dagger (†). The initial H14 hemagglutinin subtype isolates described by Kawaoka et al. (1990) are indicated with asterisks (*).

The phylogeny for the H14 subtype HA gene supported two major clades corresponding to continent of sample origin (Eurasia vs. North America) and time period of collection (1982 vs. 2010–2013; Figure 1). Topology provided evidence that the HA genes from isolates from Guatemala and Texas were derived from a H14 lineage that may have circulated in wild ducks in the Mississippi Flyway of North America in 2010 (Figure 1). Phylogenies for internal gene segments supported North American ancestry for isolates from Guatemala and Texas at the PB2, PB1, PA, NP, M, and NS gene segments (Figure 2, Figures S1–S6). Excluding the H14 HA subtype isolates from the former Soviet Union, only two H14 isolates, A/long-tailed duck/Wisconsin/10OS4225/2010(H14N6) and A/mallard/Wisconsin/10OS3941/2010(H14N6), had Eurasian lineage gene segments (Figure 2, Figures S1–S6). Each of these isolates had a NS gene segment of Eurasian ancestry (Figure 2, Figures S1–S6) and the two were identical with regard to nucleotide similarity (Table S2). None of the internal gene segments for H14 HA subtype IAVs were inferred as being of South American ancestry. The NA genes from all three isolates from Guatemala and Texas shared high identity (≥98%) with isolates derived from wild ducks sampled in the central United States (Table 1).

**Table 1:** Highest nucleotide identity for neuramindase genes from H14 hemagglutinin subtype influenza A virus strains isolated from blue-winged teal in Guatemala and Texas as identified using the NCBI BLAST function.

strain^1^	top BLAST result^1^	GenBank accession	identity
A/BWTE/GT/CIP049H105-15/2011(H14N3)	A/NSHO/MO/10OS4750/2010(H7N3)	CY133418	98%
A/BWTE/GT/CIP049H106-62/2011(H14N6)	A/BWTE/ND/AI09-4039/2009(H3N6)	CY140921	99%
A/BWTE/TX/AI13-1028/2013(H14N5)	A/NSHO/MS/09OS025/2009(H12N5)	CY079390	98%

1 Abbreviations have been used in strain names for host species (BWTE = blue-winged teal, NSHO = northern shoveler) and sample locations (GT = Guatemala, MO = Missouri, MS = Mississippi, ND = North Dakota, TX = Texas).

### Experimental Challenge

There was no evidence of IAV infection in the three sham-inoculated mallards during the trial. All pre- and post-inoculation serum samples were negative for antibodies to AI virus by the bELISA and VN; all oropharyngeal and cloacal swab samples were negative by virus isolation; and no NP antigen was identified with immunohistochemistry in tissues of the sham-inoculated mallard necropsied on 2 DPI.

**Figure 3:**
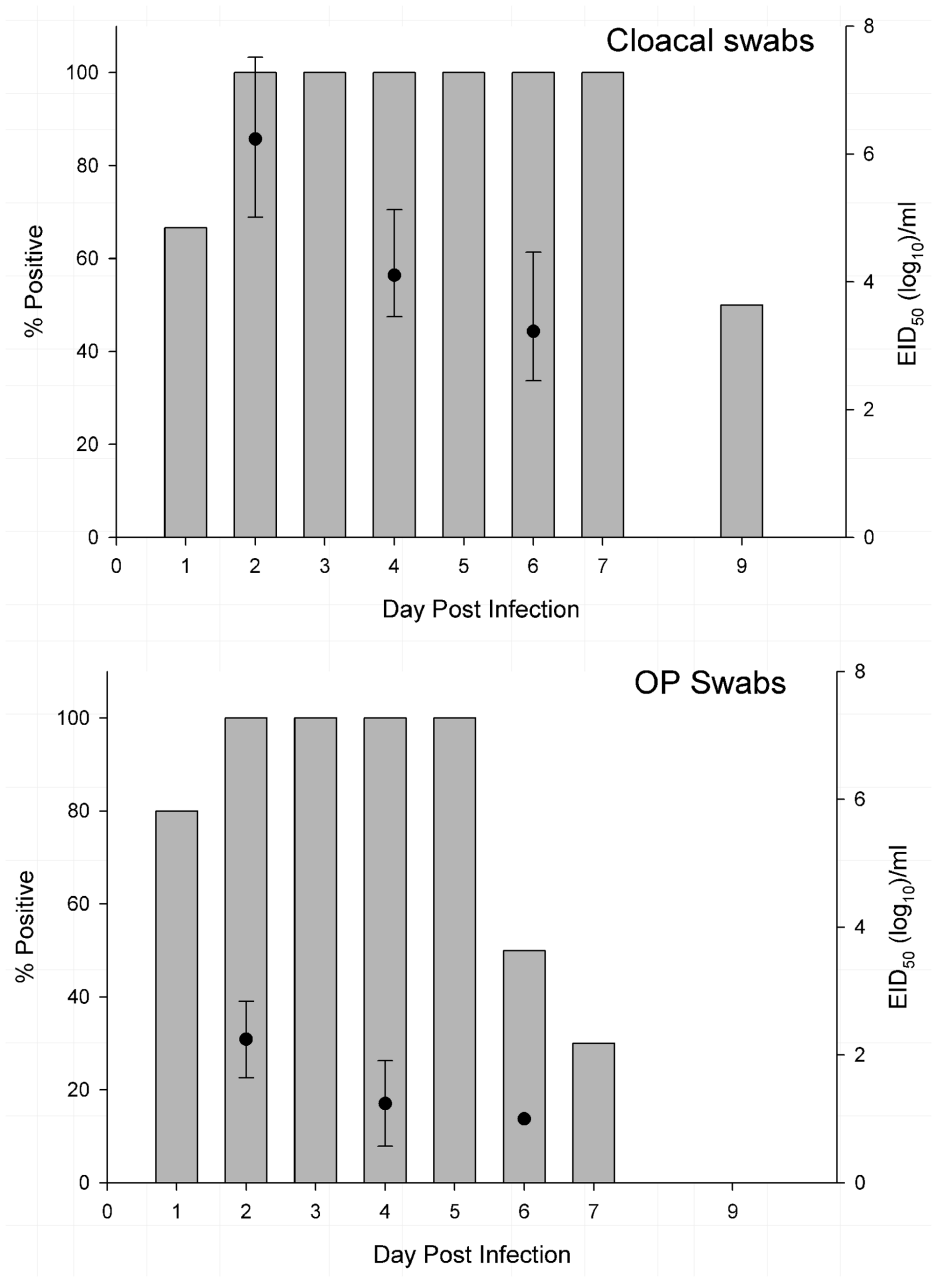
Oropharyngeal and cloacal viral shedding in six mallards intranasally inoculated with H14N5 influenza A virus (A/blue-winged teal/Texas/AI13-1028(H14N5)). Two mallards were euthanized and necropsied at 2 days post-inoculation (DPI), thus shedding data from 3 DPI on is based on four mallards. Gray bars represent the percentage of mallards that were virus isolation positive on each sampling day (left Y-axis). Black points on 2, 4, and 6 DPI represent the mean viral titers with 95% confidence intervals (right Y-axis). Virus isolation positive swab samples that had a concentration of virus below the detectable limit of the titration assay were listed as 10^1^ log_10_ EID_50_/mL.

**Figure 4:**
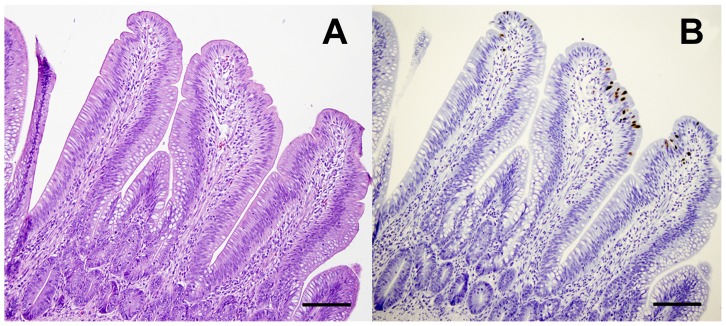
Photomicrographs of the ileum of a mallard intranasally inoculated with H14N5 influenza A virus (A/blue-winged teal/Texas/AI13-1028(H14N5)) at 2 days post-inoculation. A. No microscopic lesions are present in the mucosal villi (hematoxylin and eosin stain, bar = 100 µm). B. Serial section of the ileum showing immunolabeling for avian influenza virus nucleoprotein (brown) in epithelial cells lining the tips of the villi and rarely within cells of underlying the lamina propria (Immunoperoxidase labeling, hematoxylin counterstain, bar = 100 µm).

No clinical signs of disease were observed in any of the H14N5-inoculated or contact-exposed mallards during the trial. No gross or microscopic lesions were identified in the H14N5-inoculated and contact-exposed mallards that were necropsied and examined at 2 DPI/DPC. All six of the H14N5-inoculated mallards became infected based on viral shedding and seroconversion. Pre-inoculation serum samples collected from all six H14N5-inoculated mallards were negative for antibodies to AI virus by the bELISA and VN test. Post-inoculation serum samples collected from the two H14N5-inoculated mallards euthanized at 2 DPI were negative on both serologic tests. All post-inoculation serum samples collected at 14 DPI from the remaining four H14N5-inoculated mallards were positive by the VN assay (titer range 40–160); however, only two of these four samples were positive on the bELISA. The two serum samples collected from mallards at 14 DPI that were VN positive and bELISA negative had S/N values of 0.59 and 0.68, close to the diagnostic threshold of 0.50. Viral shedding was detected in all six H14N5-inoculated mallards starting at 1 DPI and continued until 7 to 9 DPI (Figure 3). Viral titers in oropharyngeal and cloacal swabs collected at 2 DPI were higher than samples collected at 4 and 6 DPI. In all H14N5-inoculated mallards, the duration of viral shedding and viral titers were higher in the cloacal swabs than in oropharyngeal swabs. Consistent with the shedding data, NP viral antigen was detected in the lower intestinal tract and bursa of Fabricius of the two H14N5-inoculated mallards necropsied on 2 DPI, but no antigen staining was identified in the remaining examined tissues. Within the intestines, NP antigen was primarily identified in enterocytes lining the tips of the villi in the ileum, ceca, and colon and rarely in macrophages in the lamina propria underlying the positive staining epithelium (Table 2; Figure 4). No viral antigen was identified in the intestinal crypts or in epithelial cells at the base of the villi. In the bursa of Fabricius, NP antigen was present in the epithelium.

**Table 2:** Distribution of influenza A virus (IAV) nucleoprotein antigen in the gastrointestinal tract and bursa of Fabricius of mallards exposed to a H14N5 IAV through intranasal inoculation or contact exposure.

Bird ID	Esophagus^1^	Proventriculus	Duodenum	Jejunum	Ileum	Ceca	Colon	Bursa
Inoculated								
32	−	−	−	−	++	+	++	+
33	−	−	−		++	+	++	++
Contact exposed								
31	−	−	−		−	−	−	−
34	−	−	−	−	++	−	−	+

Viral antigen was not identified in larynx, trachea, lung, brain, kidney, adrenal glands, pectoral skeletal muscle, heart, or liver. ^1^– = no staining; + = staining of a few cells; ++ = staining of moderate numbers of cells; +++ = staining of numerous cells.

H14N5 IAV was re-isolated from oropharyngeal and cloacal swabs collected from both contact exposed mallards. The titers of swab samples collected at 2 DPC were low, ranging from below the detectable limit of titration to 10^1.6^ EID_50_/mL for oropharyngeal swabs and below the detectable limit of titration to 10^2.6^ EID_50_/mL for cloacal swabs. Viral antigen was detected in tissues of one of the two contact-exposed mallards (Table 2), with positive staining restricted to the rare epithelial cells lining the ileum and bursa.

## Discussion

Genomic characterization provides evidence that H14 subtype IAVs isolated from waterfowl in the New World have evolved through genetic drift and genetic shift since purported introduction from Eurasia, including reassortment with viruses of different NA subtypes. Gene segments of H14 IAVs isolated from New World waterfowl during 2011–2013 shared ancestral genetic lineages with viruses previously isolated from wild birds throughout North America in contrast to the detection of interhemispherically reassorted H14 IAVs in 2010. Phylogenetic analyses and BLAST results therefore support the introduction of a partially or wholly Eurasian lineage IAV of the H14 subtype into the New World followed by extensive reassortment with viruses of North American ancestry. Characterization of the infectivity of A/blue-winged teal/Texas/AI13-1028/2013(H14N5) in mallards provides support for similarities in viral replication and shedding as has been previously described for other waterfowl-adapted, low pathogenic IAV strains in ducks. Collectively, these findings suggest that H14 IAVs have similar genomic characteristics and infectivity as compared to low pathogenic IAVs isolated from North American waterfowl [14], [31]–[35].

High nucleotide identity among individual gene segments for New World H14 IAV isolates (e.g. ≥95% shared identity among H14 HA gene segments) is indicative of evolution of viral lineages through point mutation or genetic drift. In contrast, different NA subtypes for H14 IAV strains and lower levels of identity among homologous internal gene segments are suggestive of viral evolution through reassortment or genetic shift. Genetic drift and genetic shift are hallmarks of IAV ecology [1] and therefore these results provide support for evolution of H14 subtype viruses through expected mechanisms in New World waterfowl.

Eurasian lineage NA and NS genes were reported in two H14 HA subtype IAVs isolated in 2010 [12]; however, genomic characterization provided support for all gene segments derived from H14 IAVs originating from samples collected during 2011–2013 to be genetically most closely related to low pathogenic viruses of North American ancestry. These results are consistent with global patterns in IAV evolution in wild birds. Infrequent exchange of IAV gene segments between Eurasia and North America has consistently been found in wild birds sampled at locations other than western Alaska [14], [20], [32], with allopatric speciation following introduction in the relatively rare instances in which a gene segment becomes established through selective advantage [14], [36]. Thus, the Eurasian NA and NS genes reported in isolates derived from samples in Wisconsin in 2010 may have been ‘diluted’ in the New World through time and space [32] via a combination of competition and reassortment since initial introduction of H14 IAV into North America. In contrast, a lack of population immunity in New World waterfowl to the H14 HA antigen may confer a selective advantage to viruses of this subtype and facilitate the establishment of H14 HA viral genes in the New World. If the H14 HA subtype was truly introduced into immunologically naïve New World waterfowl only relatively recently (e.g. in 2010), the frequency, geographic extent, and host range for detection of viruses of this subtypes may all continue to expand.

The results of the H14N5 infectivity trial in mallards were consistent with field and experimental data on IAV infection in mallards and domestic ducks with waterfowl-origin low pathogenic virus strains. No clinical disease or lesions were observed among mallards experimentally infected with H14N5 virus, consistent with previous studies of low pathogenic IAV infections in mallards and related breeds of domestic ducks [8], [31], [34]–[35], [37]. Similarly, titers of viral shedding for mallards challenged with the H14N5 IAV were highest at 2 DPI, consistent with peak viral titers observed in mallards 2–3 DPI when challenged with H1N2, H3N8, H4N1, H5N2 viruses [31], [34]–[35]. IAV was detected up to nine DPI in the current study using cloacal swabs, comparable to detection of viral shedding using this sample type up to 12 DPI in mallards inoculated with a H3N8 virus and 6–7 DPI in Pekin ducks inoculated with a low pathogenic H5N1 [38] and H7N2 IAVs [37]. The distribution of viral antigen among mallards infected with H14N5 IAV was primarily restricted to enterocytes of the lower intestinal tract and the epithelium lining the bursa of Fabricius, which is consistent with reported tropism of low pathogenic H2N3, H3N3, H3N8, H4N6 IAVs in naturally infected hatch year mallards [33] and commercially hatched mallards experimentally infected with H3N8 and H5N2 viruses [35].

In contrast to similarities observed between results of this study and other challenge studies in which ducks were inoculated with waterfowl-origin IAV strains, results regarding the pathogenesis, replication, and shedding of H14N5 IAV in mallards differed from those reported for experimental trials using mammal-, poultry- or gull-origin viruses as inoculum in waterfowl. Human- and swine-origin IAV strains inoculated into mallards and Pekin ducks only replicated in the upper respiratory tract of birds and cloacal shedding was not detected [31], [37]. Histopathological lesions in Pekin ducks inoculated with chicken-origin low pathogenic H5N2 and H5N3 IAV strains were detected almost exclusively in the respiratory tract and viral shedding was only detected using tracheal swabs [38]. Only one of three gull-origin H13 HA subtype IAV strains caused a detectable infection in experimentally inoculated mallards and no viral shedding was detected after 2 DPI in infected birds [39]. Furthermore, none of the H13 HA subtype virus strains were transmitted to naïve contact birds placed at 1 DPI [39]. Collectively, comparison of results between the current study and previous investigations provides evidence to suggest host-adaptation of A/blue-winged teal/Texas/AI13-1028/2013(H14N5) to waterfowl. However, this does not preclude the possibility that this virus strain could replicate in poultry or mammalian hosts as was previously shown for A/mallard/Gurjev/263/1982(H14N5) [8].

Although multiple lines of evidence suggest that H14 subtype IAVs recently detected in New World waterfowl have evolved and adapted similar to other low pathogenic viruses circulating in wild waterbirds, the full impacts of the purported emergence of this subtype in North America remains unknown. Homo- and heterospecific immunity may explain subtypic diversity [16] and seasonal patterns in the relative abundance of IAV subtypes in waterfowl [11]. Thus, the introduction and spread of the H14 IAV subtype in the New World could alter infection dynamics among viruses of different HA subtypes. Additional research on population immunity, cross protection of antibodies, and seasonal trends in the subtype diversity may clarify how changes in the frequency of H14 IAV infections could alter viral ecology among New World waterbirds. Furthermore, a previous investigation provided evidence for replication of H14 viruses in poultry and a mammalian model [8] and therefore further assessments of potential impacts to domestic animals and humans may also be warranted should detections of viruses of this subtype become more frequent or widespread.

## Supporting Information

Figure S1 Maximum likelihood phylogenetic tree showing inferred relationship among nucleotide sequences for the PB2 gene of influenza A viruses of the H14 subtype and reference isolates originating from Eurasia, North America, and South America. Bootstrap support values ≥70 are shown.
(PDF)

Figure S2 Maximum likelihood phylogenetic tree showing inferred relationship among nucleotide sequences for the PB1 gene of influenza A viruses of the H14 subtype and reference isolates originating from Eurasia, North America, and South America. Bootstrap support values ≥70 are shown.
(PDF)

Figure S3 Maximum likelihood phylogenetic tree showing inferred relationship among nucleotide sequences for the PA gene of influenza A viruses of the H14 subtype and reference isolates originating from Eurasia, North America, and South America. Bootstrap support values ≥70 are shown.
(PDF)

Figure S4 Maximum likelihood phylogenetic tree showing inferred relationship among nucleotide sequences for the NP gene of influenza A viruses of the H14 subtype and reference isolates originating from Eurasia, North America, and South America. Bootstrap support values ≥70 are shown.
(PDF)

Figure S5 Maximum likelihood phylogenetic tree showing inferred relationship among nucleotide sequences for the M gene of influenza A viruses of the H14 subtype and reference isolates originating from Eurasia, North America, and South America. Bootstrap support values ≥70 are shown.
(PDF)

Figure S6 Maximum likelihood phylogenetic tree showing inferred relationship among nucleotide sequences for the NS gene of influenza A viruses of the H14 subtype and reference isolates originating from Eurasia, North America, and South America. Bootstrap support values ≥70 are shown.
(PDF)

Table S1 Genbank accession numbers for reference sequences used in phylogenetic analyses of H14 hemagglutinin subtype influenza A viruses.
(XLSX)

Table S2 Nucleotide identity among homologous gene segments of H14 hemagglutinin subtype influenza A viruses, 1982–2013.
(XLSX)

## References

[1] Webster RG, Bean WJ, Gorman OT, Chambers TM, Kawaoka Y (1992) Evolution and ecology of influenza A viruses. Microbiol Rev 56: 152–179. 1579108 10.1128/mr.56.1.152-179.1992 PMC372859

[2] Röhm C, Zhou N, Süss J, Mackenzie J, Webster RG (1996) Characterization of a novel influenza hemagglutinin, H15: criteria for determination of influenza A subtypes. Virology 217: 508–516. 8610442 10.1006/viro.1996.0145

[3] Fouchier RAM, Munster V, Wallensten A, Bestebroer TM, Herfst S, et al (2005) Characterization of a novel influenza A virus hemagglutinin subtype (H16) obtained from black-headed gulls. J Virol 79: 2814–2822. 15709000 10.1128/JVI.79.5.2814-2822.2005 PMC548452

[4] Hinshaw VS, Air GM, Gibbs AJ, Graves L, Prescott B, et al (1982) Antigenic and genetic characterization of a novel hemagglutinin subtype of influenza A viruses from gulls. J Virol 42: 865–872. 7097861 10.1128/jvi.42.3.865-872.1982 PMC256920

[5] Kawaoka Y, Chambers TM, Sladen WL, Webster RG (1988) Is the gene pool of influenza viruses in shorebirds and gulls different from that in ducks? Virology 163: 247–250. 3348002 10.1016/0042-6822(88)90260-7

[6] Stallknecht D (1997) Ecology and epidemiology of avian influenza viruses in wild bird populations: waterfowl, shorebirds, pelicans, cormorants, etc. Avian Dis 47: 61–69.

[7] Liu S, Ji K, Chen J, Tai D, Jiang W, et al. (2009) Panorama phylogenetic diversity and distribution of type A influenza virus. PLoS ONE 4: e5022. Available: http://www.plosone.org/article/info%3Adoi%2F10.1371%2Fjournal.pone.0005022. Accessed 2009 Mar 30. 10.1371/journal.pone.0005022 PMC2658884 19325912

[8] Kawaoka Y, Yamnikova S, Chambers TM, Lvov DK, Webster RG (1990) Molecular characterization of a new hemagglutinin, subtype H14, of influenza A virus. Virology 179: 759–767. 2238469 10.1016/0042-6822(90)90143-f

[9] Nolting J, Fries AC, Slemons RD, Courtney C, Hines N, et al. (2012) Recovery of H14 influenza A virus isolates from sea ducks in the Western Hemisphere. PLOS Currents Influenza. Available: http://www.ncbi.nlm.nih.gov/pmc/articles/PMC3269293.1/. Accessed 2013 Dec 4. doi:10.1371/currents.RRN1290. 10.1371/currents.RRN1290 PMC3269293 22307173

[10] Boyce WM, Schobel S, Dugan VG, Halpin R, Lin X, et al. (2013) Complete genome sequence of a reassortant H14N2 avian influenza virus from California. Genome Announcements 1: e00543–13. Available: http://genomea.asm.org/content/1/4/e00543-13.full.pdf+html. Accessed 2013 Dec 10. 10.1128/genomeA.00543-13 PMC3731840 23908286

[11] Ramey AM, Poulson RL, González-Reiche AS, Wilcox BR, Walther P, et al. (2013) Evidence for seasonal patterns in the relative abundance of avian influenza virus subtypes in blue-winged teal (Anas discors). J Wildl Dis. In Press. 10.7589/2013-09-232 24949926

[12] Fries AC, Nolting JM, Danner A, Webster RG, Bowman AS, et al. (2013) Evidence for the circulation and inter-hemispheric movement of the H14 subtype influenza A virus. PLoS ONE 8: e59216. Available: http://www.plosone.org/article/info%3Adoi%2F10.1371%2Fjournal.pone.0059216. Accessed 2013 Apr 2. 10.1371/journal.pone.0059216 PMC3610705 23555632

[13] Bellrose FC (1980) Ducks, geese, and swans of North America. Harrisburg: Stackpole Books. 568 p.

[14] Olsen B, Munster VJ, Wallensten A, Waldenström J, Osterhaus ADME, et al (2006) Global patterns of influenza A virus in wild birds. Science 312: 384–388. 16627734 10.1126/science.1122438

[15] Pearce JM, Reeves AB, Ramey AM, Hupp JW, Ip HS, et al (2011) Interspecific exchange of avian influenza virus genes in Alaska: the influence of trans-hemispheric migratory tendency and breeding ground sympatry. Mol Ecol 20: 1015–1025. 21073586 10.1111/j.1365-294X.2010.04908.x PMC3041836

[16] Latorre-Margalef N, Grosbois V, Wahlgren J, Munster VJ, Tolf C, et al. (2013) Heterosubtypic immunity to influenza A virus infections in mallards may explain existence of multiple virus subtypes. PLoS Pathog 9: e1003443. Available: http://www.plospathogens.org/article/info%3Adoi%2F10.1371%2Fjournal.ppat.1003443. Accessed 2013 Jun 23. 10.1371/journal.ppat.1003443 PMC3688562 23818849

[17] Höper D, Hoffmann B, Beer M (2011) A comprehensive deep sequencing strategy for full-length genomes of influenza A. PLoS ONE 6: e19075. Available: http://www.plosone.org/article/info%3Adoi%2F10.1371%2Fjournal.pone.0019075. Accessed 2013 Dec 13. 10.1371/journal.pone.0019075 PMC3084732 21559493

[18] Djikeng A, Halpin R, Kuzmickas R, Depasse J, Feldblyum J, et al. (2008) Viral genome sequencing by random priming methods. BMC Genomics 9: 5. Available: http://www.biomedcentral.com/1471-2164/9/5/. Accessed 2013 Dec 13. 10.1186/1471-2164-9-5 PMC2254600 18179705

[19] Kimble JB, Angel M, Wan H, Sutton TC, Finch C, et al. (2013). Alternative reassortment events leading to transmissible H9N1 influenza viruses in the ferret model. J Virol doi:10.1128/JVI.02677-13. 10.1128/JVI.02677-13 PMC3911707 24131710

[20] Ramey AM, Pearce JM, Flint PL, Ip HS, Derksen DV, et al (2010) Intercontinental reassortment and genomic variation of low pathogenic avian influenza viruses isolated from northern pintails (Anas acuta) in Alaska: Examining the evidence through space and time. Virology 401: 179–189. 20227102 10.1016/j.virol.2010.02.006

[21] Zou S (1997) A practical approach to genetic screening for influenza virus variants. J Clinical Microbiol 35: 2623–2627. 9316919 10.1128/jcm.35.10.2623-2627.1997 PMC230022

[22] Hoffmann E, Stech J, Guan Y, Webster RG, Perez DR (2001) Universal primer set for the full length amplification of all influenza A viruses. Arch Virol 146: 2275–2289. 11811679 10.1007/s007050170002

[23] Phipps LP, Essen SC, Brown IH (2004) Genetic subtyping of influenza A viruses using RT-PCR with a single set of primers based on conserved sequences within the HA2 coding region. J Virol Methods 122: 119–122. 15488629 10.1016/j.jviromet.2004.08.008

[24] Bragstad K, Jørgensen PH, Handberg KJ, Mellergaard S, Corbet S, Fomsgaard A (2005) New avian influenza A virus subtype combination H5N7 identified in Danish mallard ducks. Virus Res 109: 181–190. 15763149 10.1016/j.virusres.2004.12.004

[25] Obenauer JC, Denson J, Mehta PK, Su X, Mukatira S, et al (2006) Large-scale sequence analysis of avian influenza isolates. Science 311: 1562–1563. 16439620 10.1126/science.1121586

[26] Li OTW, Barr I, Leung CYH, Chen H, Guan Y, et al (2007) Reliable universal RT-PCR assays for studying influenza polymerase subunit gene sequences from all 16 haemagglutinin subtypes. J Virol Methods 142: 218–222. 17324474 10.1016/j.jviromet.2007.01.015

[27] Tamura K, Peterson D, Peterson N, Stecher G, Nei M, et al (2011) MEGA5: Molecular evolutionary genetics analysis using maximum likelihood, evolutionary distance, and maximum parsimony methods. Mol Biol Evol 28: 2731–2739. 21546353 10.1093/molbev/msr121 PMC3203626

[28] Swayne DE, Senne DA, Suarez DL (2008) Influenza. In: Dufour-Zavala L, Swayne DE, Glisson JR, Pearson JE, Reed WM, et al. editors. A laboratory manual for the isolation and identification of avian pathogens, 5th Edition. American Kennett Square: Association of Avian Pathologists. 128–134.

[29] Driskell EA, Jones CA, Stallknecht DE, Howerth EW, Tompkins SM (2010) Avian influenza virus isolates from wild birds replicate and cause disease in a mouse model of infection. Virology 399: 280–289. 20123144 10.1016/j.virol.2010.01.005

[30] Reed LJ, Muench H (1938) A simple method of estimating fifty-percent endpoints. American J Hygiene 27: 493–497.

[31] Webster RG, Yakhno M, Hinshaw VS, Bean WJ, Murti G (1978) Intestinal influenza: replication and characterization of influenza virus in ducks. Virology 84: 268–278. 23604 10.1016/0042-6822(78)90247-7 PMC7131577

[32] Pearce JM, Ramey AM, Flint PL, Koehler AV, Fleskes JP, et al (2009) Avian influenza at both ends of a migratory flyway: characterizing viral genomic diversity to optimize surveillance plans for North America. Ecol Evol 2: 457–468. 10.1111/j.1752-4571.2009.00071.x PMC3352445 25567891

[33] Daoust P-V, Kibenge FSB, Fouchier RAM, van de Bildt MWG, van Riel D, et al (2011) Replication of low pathogenic avian influenza virus in naturally infected mallard ducks (Anas platyrhynchos) causes no morphological lesions. J Wildl Dis 47: 401–409. 21441193 10.7589/0090-3558-47.2.401

[34] Brown JD, Berghaus RD, Costa TP, Poulson R, Carter DL, et al (2012) Intestinal excretion of a wild bird-origin H3N8 low pathogenic avian influenza virus in mallards (*Anas plytrhynchos*). J Wildl Dis 48: 991–998. 23060500 10.7589/2011-09-280 PMC11373667

[35] França M, Stallknecht DE, Poulson R, Brown J, Howerth EW (2012) The pathogenesis of low pathogenic avian influenza in mallards. Avian Dis 56: 976–980. 23402122 10.1637/10153-040812-ResNote.1 PMC11361407

[36] Bahl J, Vijaykrishna D, Holmes EC, Smith GJD, Guan Y (2009) Gene flow and competitive exclusion of avian influenza A virus in natural reservoir hosts. Virology 390: 289–297. 19501380 10.1016/j.virol.2009.05.002 PMC2753668

[37] Kida H, Yanagawa R, Matsuoka Y (1980) Duck influenza lacking evidence of disease signs and immune response. Infect Immun 30: 547. 7439994 10.1128/iai.30.2.547-553.1980 PMC551346

[38] Mundt E, Gay L, Jones L, Saavedra G, Tompkins SM, et al (2009) Replication and pathogenesis associated with H5N1, H5N2, and H5N3 low-pathogenic avian influenza virus infection in chickens and ducks. Arch Virol 154: 1241–1248. 19575275 10.1007/s00705-009-0437-2

[39] Brown JD, Poulson R, Carter DL, Lebarbenchon C, Pantin-Jackwood M, et al (2012) Susceptibility of avian species to North American H13 low pathogenic avian influenza viruses. Avian Dis 56: 969–975. 23402121 10.1637/10158-040912-Reg.1 PMC11331429

