# Supplementary figures and images for: Genomic Characterization of H14 Subtype Influenza A Viruses in New World Waterfowl and Experimental Infectivity in Mallards (Anas platyrhynchos)
Source: PLoS One. 2014 May 1;9(5):e95620. doi: 10.1371/journal.pone.0095620 (PMC4006863; doi:10.1371/journal.pone.0095620)

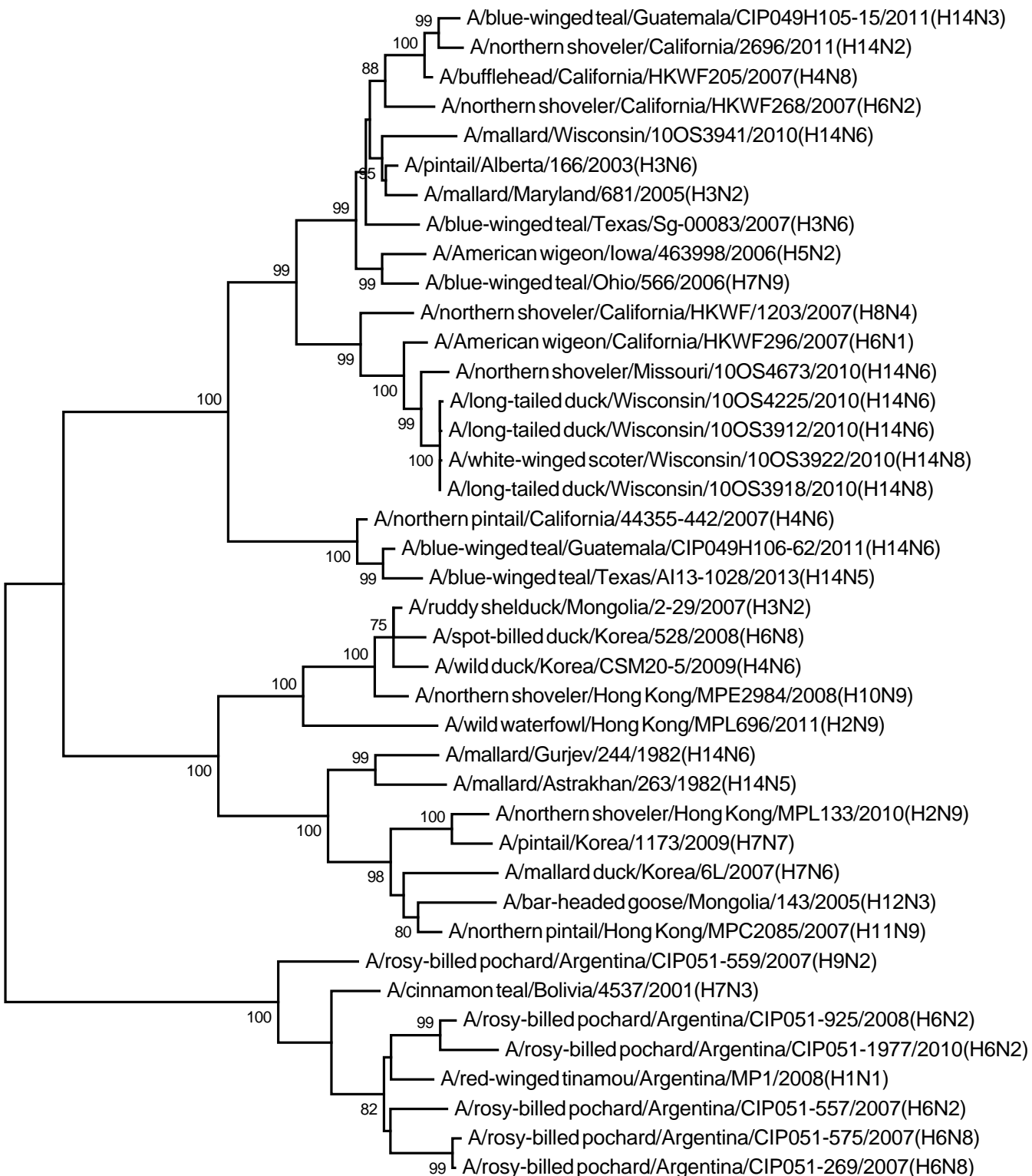

Supplement: Figure S1 — Maximum likelihood phylogenetic tree showing inferred relationship among nucleotide sequences for the PB2 gene of influenza A viruses of the H14 subtype and reference isolates originating from Eurasia, North America, and South America. Bootstrap support values ≥70 are shown. (PDF) [file pone.0095620.s001.pdf]

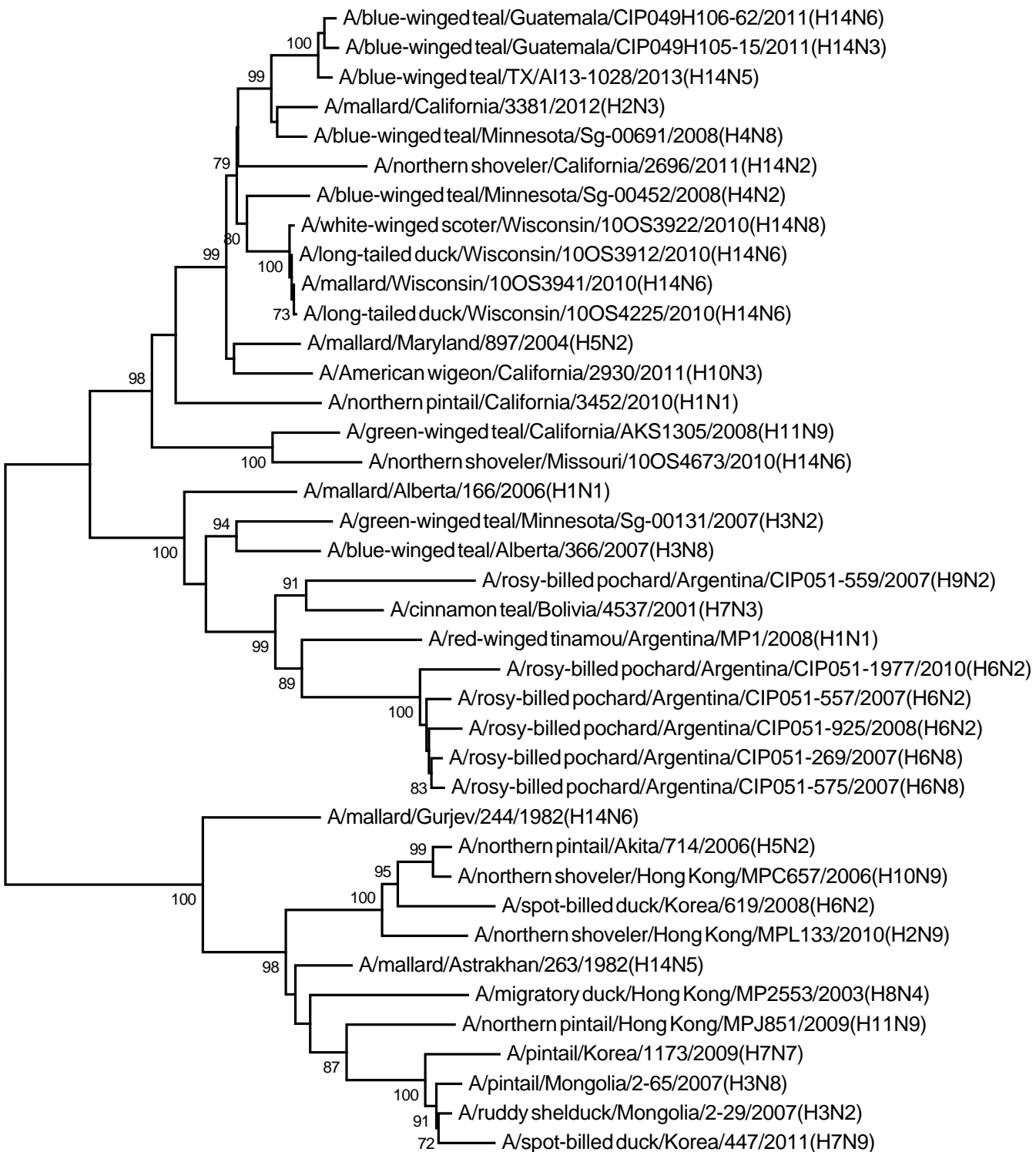

Supplement: Figure S2 — Maximum likelihood phylogenetic tree showing inferred relationship among nucleotide sequences for the PB1 gene of influenza A viruses of the H14 subtype and reference isolates originating from Eurasia, North America, and South America. Bootstrap support values ≥70 are shown. (PDF) [file pone.0095620.s002.pdf]

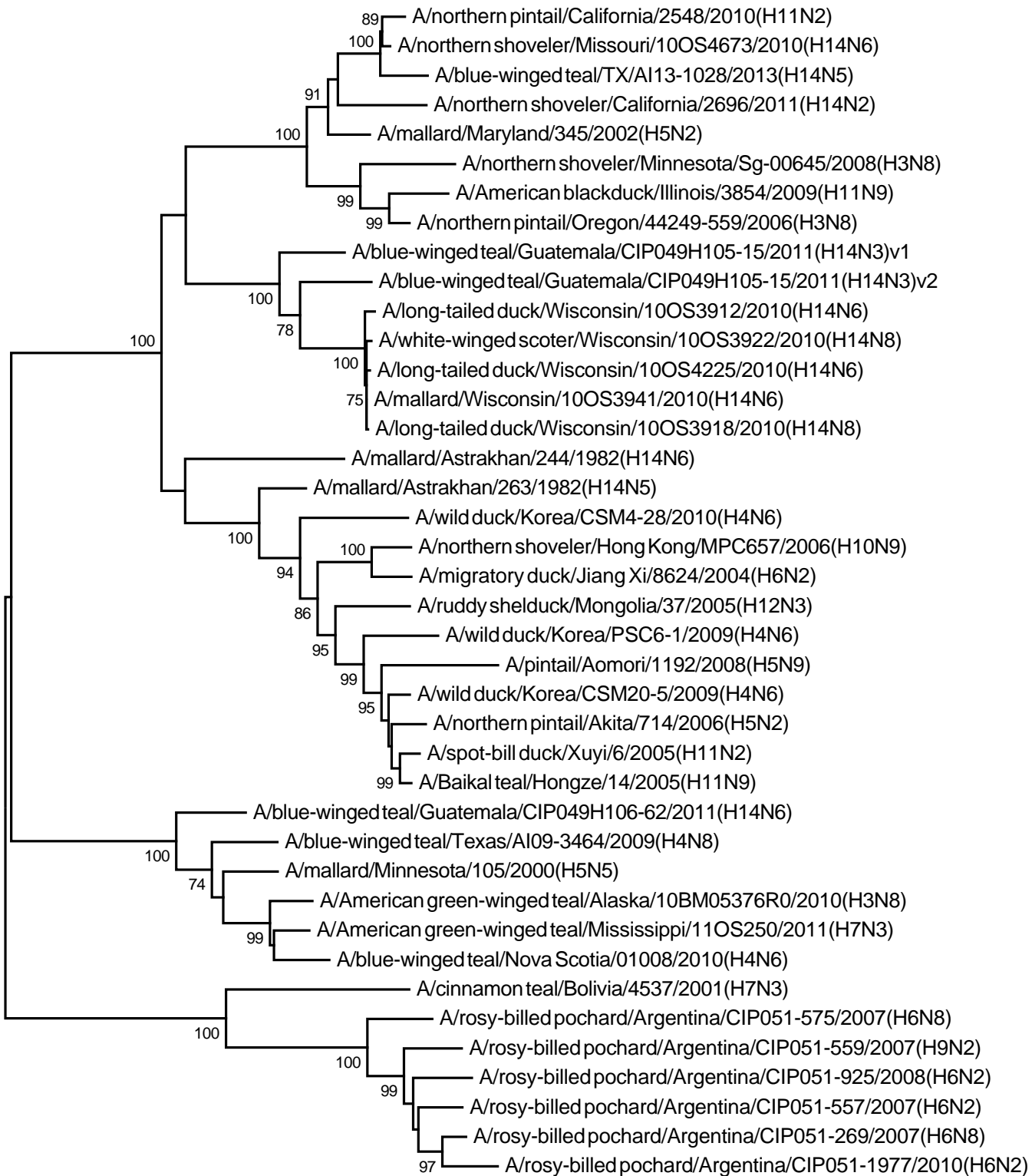

Supplement: Figure S3 — Maximum likelihood phylogenetic tree showing inferred relationship among nucleotide sequences for the PA gene of influenza A viruses of the H14 subtype and reference isolates originating from Eurasia, North America, and South America. Bootstrap support values ≥70 are shown. (PDF) [file pone.0095620.s003.pdf]

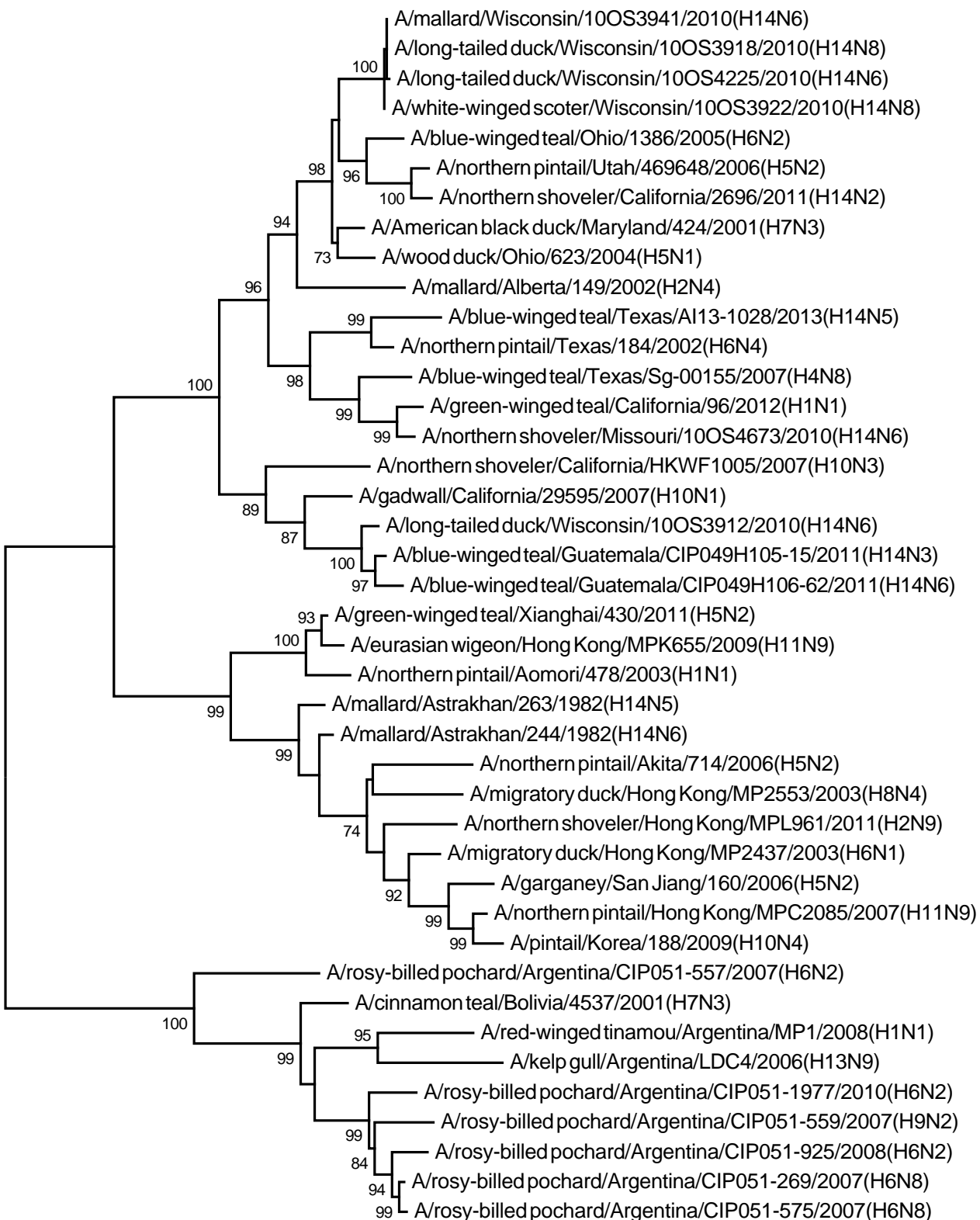

Supplement: Figure S4 — Maximum likelihood phylogenetic tree showing inferred relationship among nucleotide sequences for the NP gene of influenza A viruses of the H14 subtype and reference isolates originating from Eurasia, North America, and South America. Bootstrap support values ≥70 are shown. (PDF) [file pone.0095620.s004.pdf]

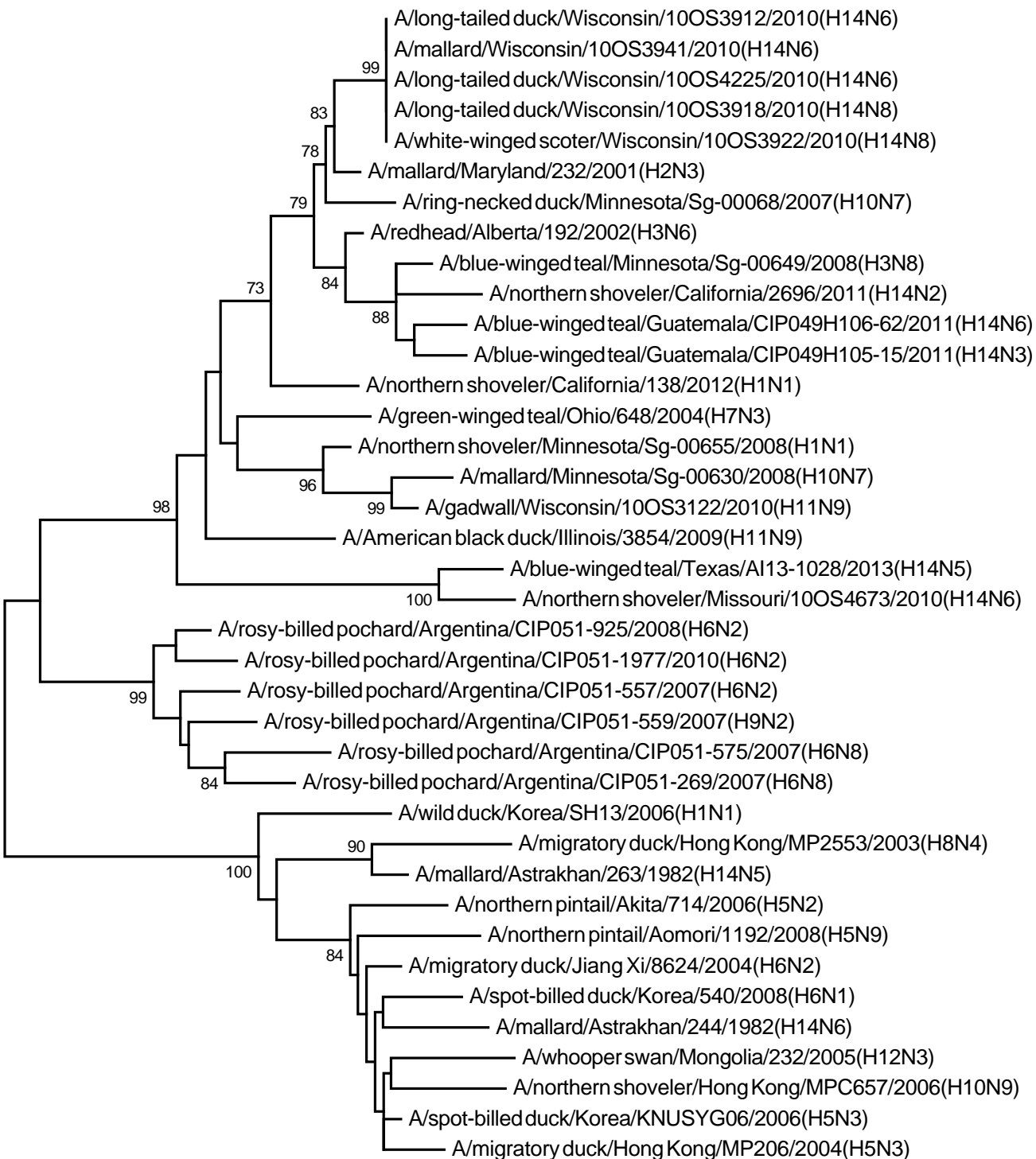

Supplement: Figure S5 — Maximum likelihood phylogenetic tree showing inferred relationship among nucleotide sequences for the M gene of influenza A viruses of the H14 subtype and reference isolates originating from Eurasia, North America, and South America. Bootstrap support values ≥70 are shown. (PDF) [file pone.0095620.s005.pdf]

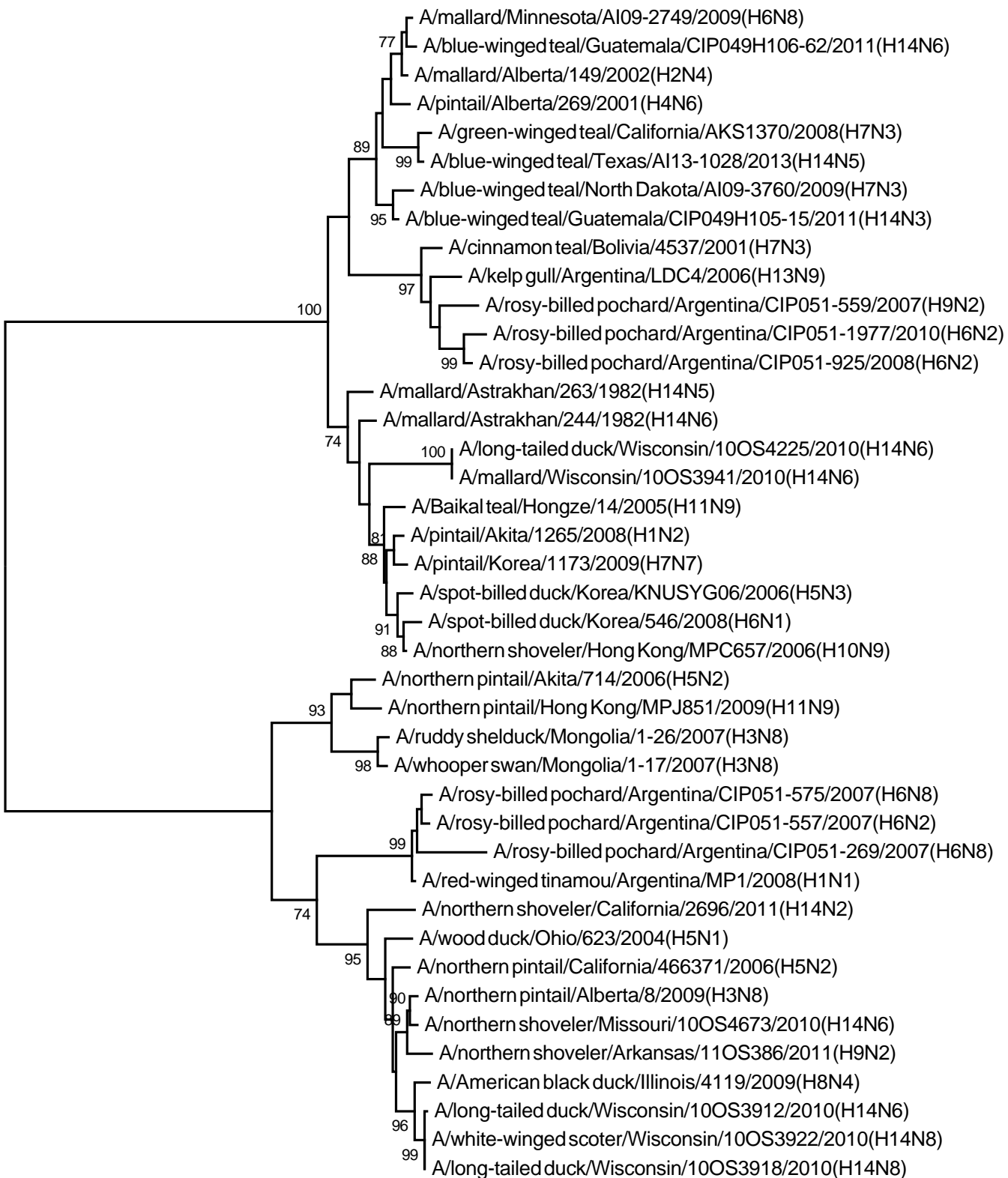

Supplement: Figure S6 — Maximum likelihood phylogenetic tree showing inferred relationship among nucleotide sequences for the NS gene of influenza A viruses of the H14 subtype and reference isolates originating from Eurasia, North America, and South America. Bootstrap support values ≥70 are shown. (PDF) [file pone.0095620.s006.pdf]
